# Improvements in Simultaneous Sodium and Calcium Imaging

**DOI:** 10.3389/fncel.2018.00514

**Published:** 2019-01-08

**Authors:** Kenichi Miyazaki, John E. Lisman, William N. Ross

**Affiliations:** ^1^Department of Physiology, New York Medical College, Valhalla, NY, United States; ^2^Marine Biological Laboratory, Woods Hole, MA, United States; ^3^Department of Biology, Brandeis University, Waltham, MA, United States

**Keywords:** sodium, calcium, imaging, dendrite, spine, pyramidal neuron, CCD camera

## Abstract

High speed imaging of ion concentration changes in neurons is an important and growing tool for neuroscientists. We previously developed a system for simultaneously measuring sodium and calcium changes in small compartments in neurons (Miyazaki and Ross, 2015). We used this technique to analyze the dynamics of these ions in individual pyramidal neuron dendritic spines (Miyazaki and Ross, 2017). This system is based on high speed multiplexing of light emitting diodes (LEDs) and classic organic indicators. To improve this system we made additional changes, primarily incorporating lasers in addition to the LEDs, more sophisticated imaging protocols, and the use of newer sodium and calcium indicators. This new system generates signals with higher signal to noise ratio (S/N), less background fluorescence, and less photodynamic damage. In addition, by using longer wavelength indicators instead of indicators sensitive in the UV range, it allows for the incorporation of focal uncaging along with simultaneous imaging, which should extend the range of experiments.

## Introduction

High speed imaging of ion concentration changes has revolutionized our understanding of many aspects of nervous system function. Before the use of imaging most neuronal activity was recorded using electrodes that detected responses at one site on the neuron, usually the cell body. With imaging, using appropriate indicator molecules, activity can be recorded from many sites, including sites on neurons that are too small or distant, like dendrites or axons, to be penetrated with electrodes. Most progress has occurred using calcium indicators because these are easy to design with appropriate sensitivity and because physiological [Ca^2+^]_i_ changes are large compared to the resting [Ca^2+^]_i_ in the cell. Experiments using this class of indicator have examined the direct physiological role of [Ca^2+^]_i_ changes in different parts of the cell (e.g., calcium entry in presynaptic terminals, calcium entry through postsynaptic receptors, and calcium release from stores in the soma and dendrites). They also used the detected [Ca^2+^]_i_ changes as an indicator of electrical activity because action potentials almost always cause measurable [Ca^2+^]_i_ changes in all parts of the cell where they propagate. Some experiments have used voltage sensitive dyes (VSDs) to measure electrical activity directly in small compartments, with some experiments detecting subthreshold potential changes in dendritic spines (Popovic et al., 2015; Acker et al., 2016).

Sodium sensitive indicators have also been used to report on the activity of neurons in slices, but with less frequency, in part, because they are relatively less sensitive than calcium indicators, making the measurements more challenging. Nevertheless, there are kinds of experiments where they might be preferred, for example, when trying to detect sodium entry through AMPA receptors, which are not permeable to calcium in most pyramidal neurons. In some ways these kinds of measurements are complementary to calcium measurements, giving additional useful information about synaptic activity and electrical function.

Recently, we developed a method (Miyazaki and Ross, 2015) to simultaneously image [Ca^2+^]_i_ and [Na^+^]_i_ changes in neurons at high speed and high spatial resolution. It has some resemblance to other approaches to measure two parameters simultaneously (Vogt et al., 2011; Lee et al., 2012), but optimized to detect these two ions. We were able to combine this system with other improvements to detect and analyze these changes in individual dendritic spines on hippocampal pyramidal neurons with sufficient time resolution to follow the physiological calcium ([Ca^2+^]_i_) and sodium ion concentration ([Na^+^]_i_) changes in those structures (Miyazaki and Ross, 2017). These measurements were the first to use high speed sodium imaging with single spine resolution. Even with this success, however, we thought it would be useful to improve the system to: (a) increase the signal to noise ratio (S/N) of these measurements to reveal additional properties about spines; (b) to decrease background fluorescence to improve the accuracy of the interpretation of the fluorescence measurements in terms of [Na^+^]_i_ changes; (c) to reduce the photodynamic damage caused by the fluorescence excitation light to allow for longer experiments; and (d) to allow for the incorporation of uncaging stimulation in addition to electrical stimulation. To this end, we made several improvements to the apparatus. In this article we review the original setup for simultaneous sodium and calcium imaging, pointing out the novel aspects of the system, and we describe the new components, which are responsible for the improvements. We also indicate a few additional changes that we hope to incorporate in the near future.

## Materials and Methods

Hippocampal transverse slices were prepared from 2- to 4-week-old Sprague-Dawley rats using techniques regularly followed in our laboratory (Miyazaki and Ross, 2017). All procedures were approved by institutional IACUC committees at New York Medical College and the Marine Biological Laboratory. After incubation, individual slices were placed on the stage of an Olympus BX50WI microscope and viewed with a 20× or 60× water immersion lens. Pyramidal neurons were patched on the soma and filled with combinations of sodium and calcium indicators [either SBFI and Oregon Green Bapta-1 (OGB-1; or OGB-5N), or bis-fura-2 and ANG-2]. Some trial experiments (Figure 7) used Cal-630 as the calcium indicator. Synaptic activation was through an external theta-glass electrode placed on the slice ~10 μm from a dendrite. Fluorescence illumination, through the usual rear fluorescence port of the microscope, was provided by one of several light emitting diodes (LEDs; Miyazaki and Ross, 2015). High speed movies of the fluorescence changes from the indicators were detected by a RedShirtImaging NeuroCCD-SMQ camera (80 × 80 pixel resolution). Data taking was under the control of Neuroplex software supplied by RedShirtImaging. Analysis was under control of a package of programs, SCANDATA, also written in MATLAB. More details are provided in the previous articles (Miyazaki and Ross, 2015, 2017). Newer methods are described below.

**Figure 1:**
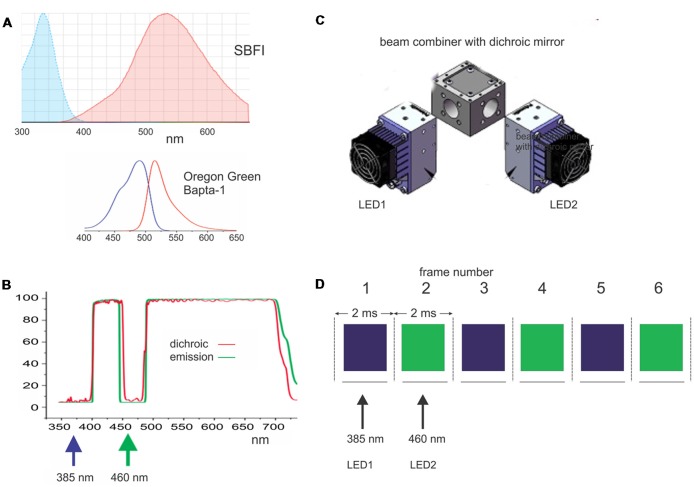
Components of the apparatus used to make simultaneous sodium and calcium measurements with light emitting diode (LED) excitation sources. **(A)** Excitation and emission spectra of SBFI (sodium indicator) and Oregon Green Bapta-1 (OGB-1; calcium indicator). Both sets of spectra are plotted on the same wavelength scale to show the separation of the excitation bands. **(B)** Custom dichroic and emission filters used for these two indicators. Note the notch in the filters to pass 460 nm excitation light for OGB-1. **(C)** Two LEDs (385 nm and 460 nm) together with a beam combiner with a dichroic mirror (DM) used to excite the two indicators. **(D)** Pattern of excitation and detection: every 2 ms alternate 1.8 ms LED pulses excite the indicators. The switching is synchronized to the frames of the CCD camera operating at 500 Hz. Adapted from Miyazaki and Ross (2015).

**Figure 2:**
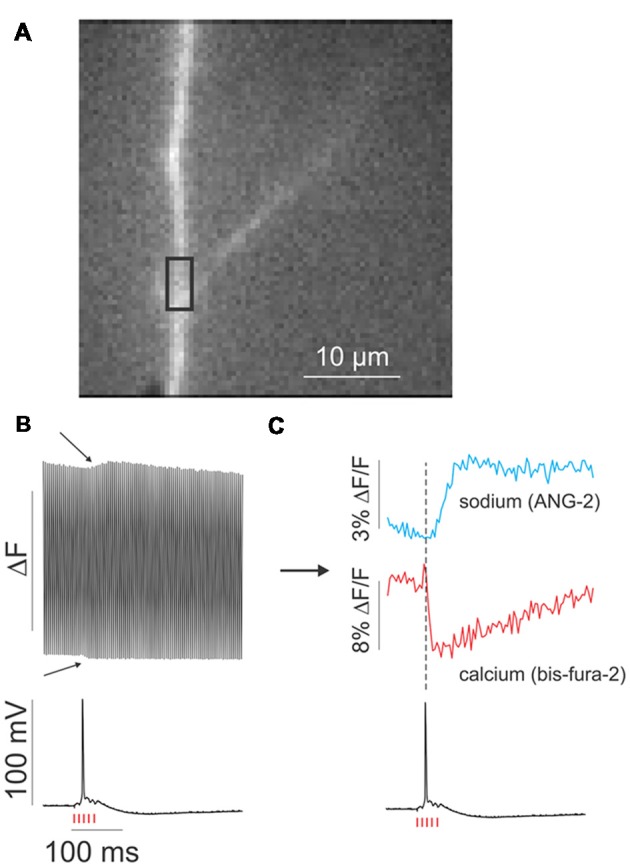
Quasi-simultaneous detection of [Ca^2+^]_i_ and [Na^+^]_i_ changes in a dendrite in response to synaptic stimulation.** (A)** Fluorescence image of a small region of an apical dendrite of a rat CA1 pyramidal neuron. A region of interest (ROI) is marked with a black rectangle. **(B)** Simultaneous fluorescence response in the ROI and the somatically measured electrical response to a train of five synaptic stimuli at 100 Hz (red hash marks). The fluorescence response switches with each frame, which are excited at alternate wavelengths. The two “bumps” (arrows) correspond to the changes in [Ca^2+^]_i_ and [Na^+^]_i_ in response to the stimulation. **(C)** Sodium and calcium signals after separating alternate frames. The calcium transient is matched to the action potential, while the sodium signal is delayed slightly. The calcium signal goes down because bis-fura-2 fluorescence decreases in response to an increase in [Ca^2+^]_i_. Adapted from Miyazaki and Ross (2015).

**Figure 3:**
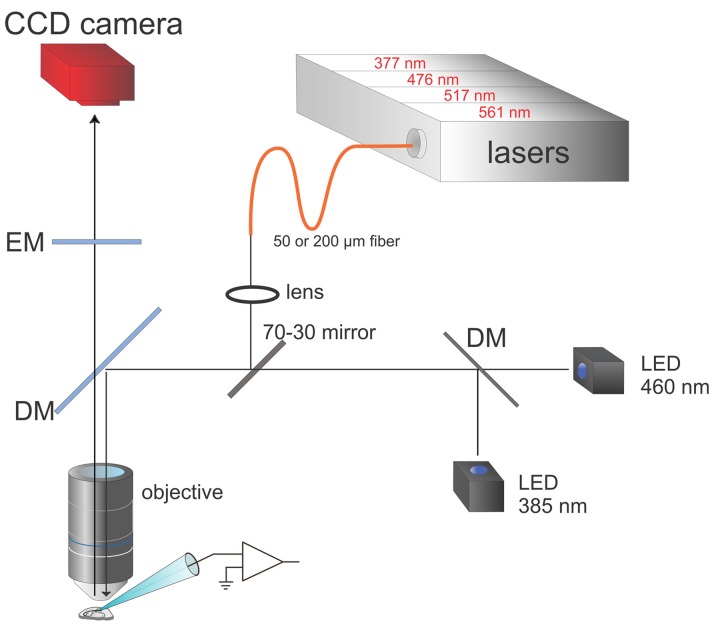
Schematic of modified apparatus including both LEDs and lasers. The box at the top contains four lasers at the indicated wavelengths. The laser outputs, which can be separately controlled, are combined and focused onto either a 50 μm or 200 μm diameter fiber optic. The face of the fiber is focused with a lens and the objective onto a region of the slice, after 30% of the light is reflected off a mirror in the microscope body and a special DM in a standard filter cube (see Figure 1B for a typical filter set). The LED outputs are combined and 70% of the light passes through to preparation. Software selects combinations of LEDs and lasers to excite the indicator dyes in the cell of interest. The CCD camera (RedShirtImaging, NeuroCCD-SMQ) detects the fluorescence changes with frames synchronized with the laser or LED pulses.

**Figure 4:**
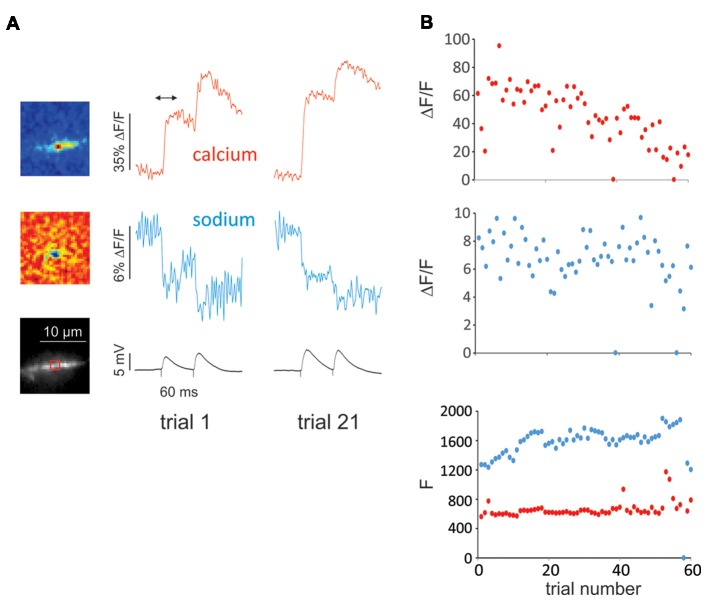
Example of experiment with many trials showing little cumulative photodynamic damage reflected in single spine sodium and calcium signals.** (A)** Pseudocolor difference images (top) and image of dendritic region with ROI indicated (bottom). The difference images show the spatial distribution of the calcium and sodium signals from the first of two synaptic stimuli (time marked by arrow above calcium signal) of the first of 60 trials. The cells were filled with 50 μM OGB-1 and 2 mM SBFI from a patch pipette on the soma. The pseudocolor images show that both changes were localized to a small region corresponding to a dendritic spine. The traces show that the signals from trial 1 and trial 21 were about the same. **(B)** Plots of peak signal sizes (ΔF/F) from the first synaptic response and resting fluorescence levels (F) for all 60 trials. The *F* values were measured at the beginning of the traces before stimulation. The peak calcium signal (ΔF/F, red dots) decreased slightly over the many trials; the peak sodium signal (ΔF/F, blue dots) showed little deterioration. In two trials (38 and 55) there was failure in both the sodium and calcium signals (an unusually small number). The *F* values stayed relatively constant until the last few trials. In four trials the resting calcium F levels were high, probably because the trials were preceded by spontaneous synaptic responses. The resting sodium *F* value did not change significantly.

**Figure 5:**
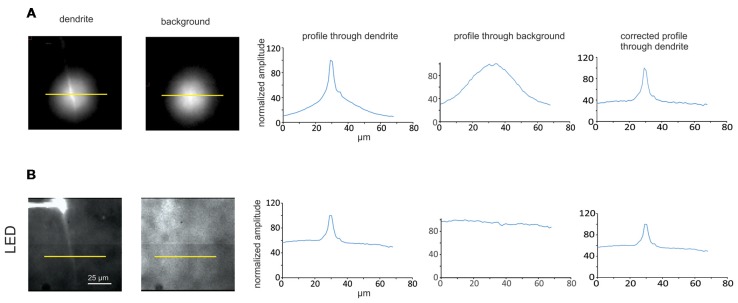
Comparison between LED and laser illumination for separating dendrite fluorescence from background. (**A**, left and middle) Images of slices with and without a dendrite in the field of view illuminated by a 377 nm laser through a 200 μm fiber and a 20× objective lens (each image has the same field of view as images in **B**). The cell was filled with 2 mM SBFI from a patch pipette on the soma. The yellow bars indicate lines along which profiles were measured. (**A**, right) Intensity profiles along yellow lines through the two images. The line through the field without the dendrite (background fluorescence) was used to normalize the profile through the dendrite, which is shown in the right trace. (**B**, left and middle) Similar images from the same neuron where the dendrite and background are illuminated by a 385 nm LED. (**B**, right) Similar profiles through the LED illuminated images. Note that the profile through the background is almost flat, which is due to the wide field illumination by the LED. Importantly, the background level using the laser illumination is less than the background when the LED is used.

**Figure 6:**
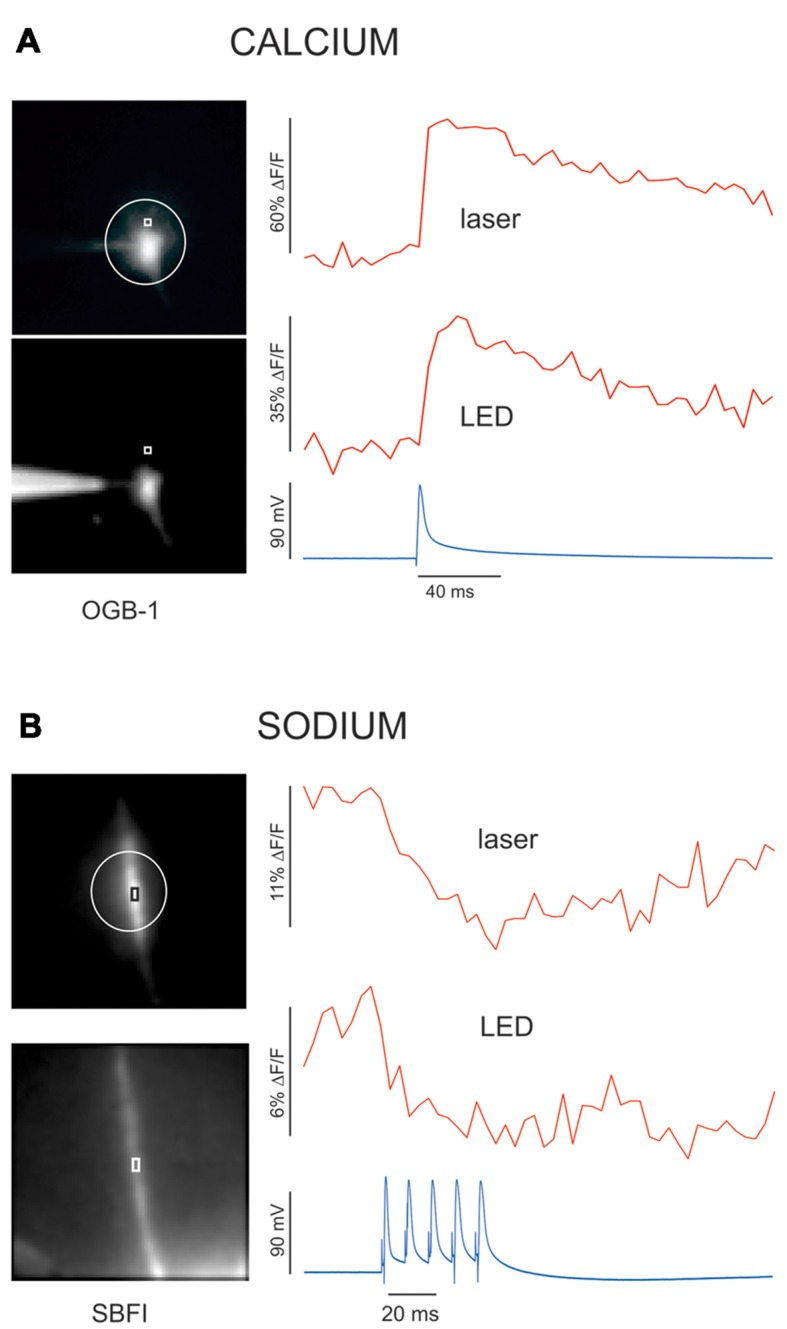
The fractional change in fluorescence (ΔF/F) is greater using a laser spot compared to when illumination is with an LED. Using a laser in one channel and an LED in the other, [Ca^2+^]_i_ or [Na^+^]_i_ changes were measured in alternate channels. The calcium signals (using OGB-1, 200 μm fiber, and 20× lens) in response to a single bAP were measured from a dendritic location in one cell **(A)** and the sodium signals (using SBFI, 200 μm fiber, and 60× lens) in response to five APs were measured from the axon of another cell **(B)**. In each case the laser and LED signals were measured simultaneously in the two channels. For both the sodium and calcium signals the peak ΔF/F response was about twice as large using the laser. Resting fluorescence levels (F) were about the same.

**Figure 7:**
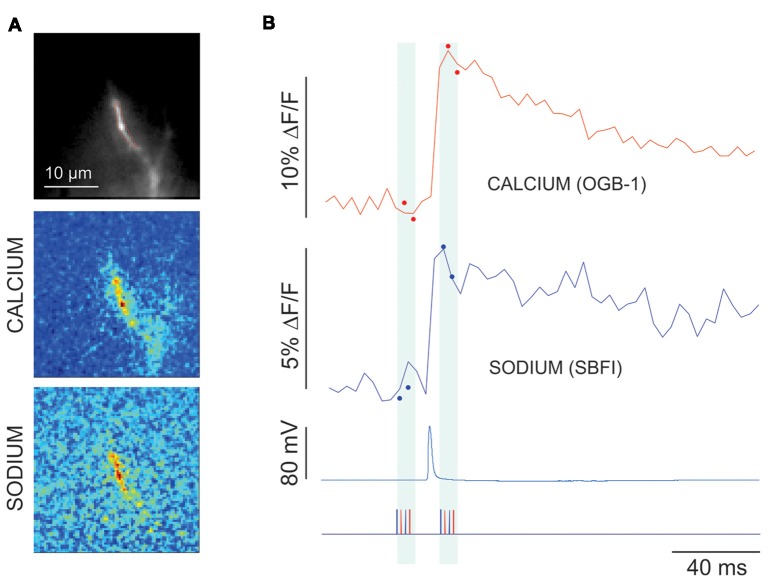
Measurement of fluorescence signals using reduced illumination protocol. **(A)** Image using 60× lens of the axon filled with 2 mM SBFI and 50 μM OGB-1, and difference images made by only illuminating four frames (two with 377 nm laser and two with 476 nm laser) before and four frames after a single intrasomatically evoked AP. **(B)** Calcium (red) and sodium (blue) signals taken by limited illumination (closed circles) and full frame illumination (solid lines) from the extended ROI in the left image. Bottom trace shows timing of the light flashes of each wavelength (blue-377 nm; red-476 nm). Sodium trace and image are inverted.

## Results

### Simultaneous Imaging Using LEDs

Figure 1 (modified from Miyazaki and Ross, 2015) shows the main features of the original system for detecting signals using LED excitation. Figure 1A shows the excitation and emission spectra for the SBFI and OGB-1 combination of sodium and calcium indicators, with the spectra aligned to the same wavelength scale. Figure 1B shows the custom filter set that allows excitation of SBFI at 385 nm and the excitation of OGB-1 at 460 nm. Figure 1C shows the arrangement of the LED illuminators with a dichroic mirror (DM) for combining the two outputs. A similar set of spectra and filter combinations are used for the bis-fura-2 and ANG-2 indicator combination (Miyazaki and Ross, 2015). Figure 1D shows the pattern of alternating 385 nm and 460 nm LED pulses aligned with the 2 ms frames of the CCD camera. This system has been upgraded slightly from the original system with the addition of a new software to generate the LED pulses, a new computer and data taking software (Turbo-SM) from RedShirtImaging, and an improved version of our analysis software (SCANDATA). We also introduced a feedback system to correct for the slight drift in LED intensity during an experimental trial due to heating of the LED modules.

Figure 2, modified from Miyazaki and Ross (2015), shows the measurement of [Na^+^]_i_ and [Ca^2+^]_i_ changes in response to a train of five synaptic stimuli. Figure 2B shows the response at a region of interest (ROI) in Figure 2A as detected by the camera. Each frame alternately records the sodium and calcium signals. When the odd numbered frames and the even numbered frames are separately plotted and aligned with the simultaneously recorded electrical response in the soma it is clear that both [Na^+^]_i_ and [Ca^2+^]_i_ changes occurred (Figure 2C). The calcium change is precisely aligned with the single synaptically evoked backpropagating action potential (bAP); the sodium signal is slightly delayed, which may be due to the slower kinetics of the NMDA receptor channel and/or sodium diffusion from its source in the spines (Miyazaki and Ross, 2017).

### Simultaneous Imaging Using Laser Spot Illumination

In addition to pursuing experiments using this system, we made some efforts to improve the simultaneous detection of sodium and calcium signals. The new components of the system are shown in Figure 3. We added a set of four laser diode modules at 377, 476, 517, and 561 nm, which are combined using DMs into one beam (Versalase, Vortran Laser Technologies). Selection among these lasers, including setting the laser pulse widths and amplitudes, is controlled by a combination of software from Vortran and a package (PULSE) written in MATLAB in our laboratory. The combined outputs of these lasers are focused onto a fiber optic patch cord (50 or 200 μm diameter). The fiber is fed into a custom side port in the fluorescence illuminator of the microscope. A 45° mirror is positioned in the object plane of the illuminator. The mirror transmits 70% of the light from the rear port from the LEDs and 30% of the laser light from the side port from the fiber. This ratio was chosen because the lasers are much brighter than the LEDs and 30% of the peak laser intensity is sufficient for most experiments. The tip of the optical fiber is focused onto the image plane of the objective, which contains the cell filled with fluorescent indicators.

We used the lasers to reduce the size of the illumination spot, to improve the S/N, to reduce background illumination, and to minimize photodynamic damage. The smallest spot the LED system can generate is about 100 μm diameter, using the built-in aperture of the Olympus fluorescence illuminator. This is because the LED is a large source and the aperture has a limited constriction range. It is possible that a custom aperture could further reduce the diameter, but it would also reduce the excitation intensity, which was already a limiting factor in obtaining good single spine signals. We did not pursue that direction. In contrast, the laser spot can be focused to a spot of 2.5 μm diameter using the 50 μm fiber optic and the 60× objective. Other illumination techniques (Popovic et al., 2015; Tanese et al., 2017) can achieve diffraction limited spots. That approach is useful for 2-photon uncaging and measurements from single spines, but is not helpful if we are trying to detect optical signals simultaneously from spines and nearby regions.

For both the LED system and the laser system we typically run the camera at 500 Hz, even though it can go faster. Five-hundred hertz is fast enough to distinguish among the time courses of physiological events without introducing additional noise due to a reduction in integration time. Typically, each LED pulse illuminates about 1.8 ms of a 2 ms camera frame; we do not illuminate during the 0.1 ms around the frame transition to prevent contamination of one frame from the light of the preceding frame. Tests confirmed that there was no cross contamination. When we use laser illumination we can make much shorter pulses (~0.1–0.4 ms) because the lasers can be much more intense than a LED; the parameter that matters is the integrated light intensity per frame. We can control this value for each laser by either varying the pulse width or the peak intensity or both. We select this value to be as high as possible without causing significant photodynamic damage.

Spot illumination with the laser also appears to reduce photodynamic damage because only a small volume of the neuron is illuminated. In our experience many more trials can be made with laser spot illumination than with widefield LED illumination using about the same excitation intensity, although it was hard to develop a rigorous way of demonstrating this conclusion. One example is shown in Figure 4, where we made over 60 measurements of single spine sodium and calcium signals with little deterioration except at the end. Illuminating the synaptic region does not appear to cause significant photodynamic damage, as assayed by the synaptic electrical response. One possible reason for this good fortune is that the indicator dyes are injected postsynaptically, while the most sensitive metabolic components are in the presynaptic terminal, which does not contain sensitizing fluorophores.

We tried to assay how much focused laser illumination reduces background fluorescence compared to using more widespread LED illumination. For this effort we filled a pyramidal neuron with 2 mM SBFI and searched for a region where a single dendrite could be isolated and in focus (Figures 5A,B, left). We measured the profile of fluorescence across the dendrite simultaneously using the 385 nm LED and laser illumination (377 nm) by recording the fluorescence from each source in different camera frames. It is not clear from these profiles how much of the fluorescence comes from the dendrite and how much from the background since the illumination along the profile is not constant and not the same for the LED and laser. To try to correct for this variation we moved to a region of the slice without a dendrite and measured the fluorescence profiles only due to the autofluorescence background of the tissue (Figures 5A,B, middle). These autofluorescence profiles should correspond to the illumination profiles of the LED and laser. We then used these profiles to normalize the intensity distribution from the images including the dendrite (Figures 5A,B, right). It is clear that after this correction the normalized intensity profile using the laser has less contribution from the background (~35%) than the background contribution to the profile (~60%) using the LED, i.e., the background contribution was reduced by ~40%. In six cells the background contribution using laser illumination was 38 ± 4% and the contribution using LED illumination was 56 ± 4% (SE), i.e., the background was 32% less using the laser. This set of measurements was made using a 200 μm diameter fiber optic, which makes a spot of about 30 μm diameter with the 20× lens. In one experiment when we used the 50 μm diameter fiber optic the background contribution to the profile using the laser was reduced slightly more (~46%). We did not do sufficient experiments with the 50 μm fiber to determine the statistical validity of this conclusion. Also, these numbers fluctuated depending on the preparation and the depth of the cell, but the lowest background was usually obtained with the 50 μm fiber.

To further compare the two illumination methods we measured sodium and calcium signals using LED and laser excitation simultaneously. In the first example (Figure 6A), we recorded the calcium signals at a dendritic location from a single bAP detected with OGB-1 using laser excitation at 476 nm and LED excitation at 460 ± 11 nm. It is clear that the S/N is better with laser excitation and the peak ΔF/F response is about twice the level obtained with the LED. In six cells we found that ΔF/F was 2.4 ± 0.2 (SE) times higher using the laser compared with ΔF/F measured using the LED. The S/N improved by the same amount, which was expected since the major source of noise is shot noise, and the intensity of the laser was adjusted to be about the same as the LED intensity in the center of the dendrite In a similar experiment we compared the sodium signals detected with SBFI from five APs in the axon initial segment region with the two excitation sources (laser at 377 nm and LED at 385 ± 8 nm) using the 60× lens which makes 10 μm diameter spot. Again (Figure 6B), it is clear that the S/N and ΔF/F values using the laser signal are better. These improvements are more than be accounted for by the reduction in background light calculated above. We are not clear about the source of the additional enhancement.

The ability to record signals using laser excitation in one channel and LED excitation in the other is useful for more than just test purposes. For some experiments it is useful to detect signals from one ion over a wide field and signals from the other ion from a spot with better S/N. For example, wide field calcium imaging can be used to identify responding spines, while focal laser excitation can improve the sodium signals from the identified spines.

### Reduction of the Illumination Duration to Reduce Photodynamic Damage

Although the results shown is Figure 4 suggest that photodynamic damage would not be a problem in many situations, there are certain experiments that might require accurate measurements over longer time periods, e.g., experiments investigating long term potentiation (LTP). Another way of limiting photodynamic damage is to reduce the integrated light per sweep. The simplest way of doing that is just to shorten the sweep as much as possible. We found that we could reduce the effective illumination period even further if we are only interested in the spatial distribution of the peak intensity of the signal and not the complete time course. For example, in an experiment to measure spike-evoked signals in the axon hillock region (Figure 7), we illuminated the preparation for a few frames just before electrical stimulation (two frames each for both wavelengths) and for a few frames after the peak of the response (Figure 7B, closed circles). We then averaged the set of frames before and after the synaptic stimulation and then subtracted one average from the other (Figure 7A, middle and bottom). This difference image corresponds to the spatial distribution of the signal maximum and only requires a few frames of illumination on each side of the response, usually less than a total of 20 ms, which is about 10% of the duration of the trials shown in the other figures in this article. Using this protocol we can record many more trials before significant photodynamic damage is observed. The weakness of this approach is that it does not record the time course of the signals. However, as shown in the figure, we can take other trials using the standard protocol to determine the time course (solid lines). This works if we expect the time course to be approximately the same in different trials. For example, some experiments examining synaptic responses might show significant variability in the amplitude of the signals, while their time courses would be expected to be more constant. Of course, this assumption would have to be tested.

### Improvements Using Long Wavelength Indicators

**Figure 8:**
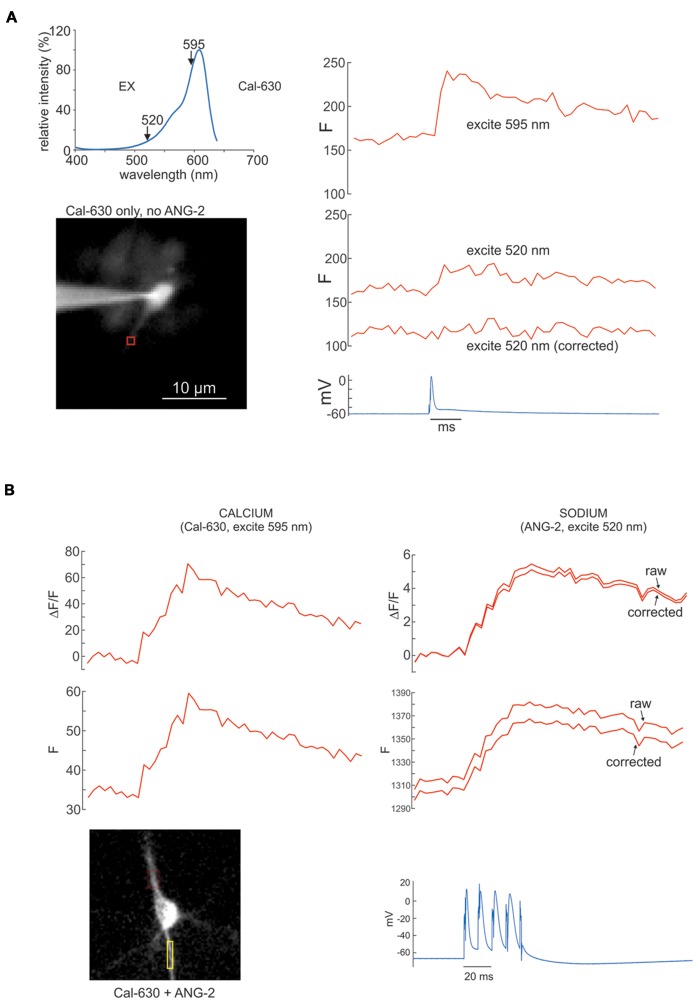
Sodium and calcium signals from APs measured with ANG-2 (sodium) and Cal-630 (calcium) with corrections for Cal-630 excitation by ANG-2 excitation light at 520 nm. (**A**, top left) Excitation spectrum of Cal-630. Note that this indicator is strongly excited at 595 nm but there is still some excitation at 520 nm. (**A**, right) Fluorescence responses from a dendritic ROI of a pyramidal neuron filled with 100 μM Cal-630 when stimulated with a single AP. The response at 595 nm is strong but there is still a signal when excited at 520 nm. The “corrected” trace, which shows no signal, was obtained when 29% of the 595 nm signal was subtracted from the 520 nm trace. (**B**, left) ΔF/F and F signals from the axon of a pyramidal neuron in response to four intrasomatically evoked APs. The cell was filled with 200 μM ANG-2 and 100 μM Cal-630, but the fluorescence only comes from the calcium indicator since exciting at 595 nm is outside the excitation band of ANG-2. (**B**, right) Similar responses to the same four APs when the cell was excited at 520 nm. The “raw” traces are the direct fluorescence signals. The “corrected” traces have the contribution of Cal-630 to the signal removed as shown in **(A)**.

A complementary approach to improving the sodium and calcium signals is to use better indicators. The best currently available sodium indicator (although not by more than a factor of two) is ANG-2 (currently sold by Ion Indicators). When we use this indicator, which is maximally excited near 513 nm, we have to use a calcium indicator that is excited away from this excitation band if we want to use our simultaneous imaging protocol. The most common calcium indicators that meet this requirement are in the fura series (most often bis-fura-2; Miyazaki and Ross, 2015). These calcium indicators are best excited near 380 nm. However, there are two disadvantages to exciting at this low wavelength. One is that excitation at this wavelength causes more background light due to autofluorescence in the tissue and more photodynamic damage because the lower wavelength photons have more energy and scatter more. Most other commonly used calcium indicators are in the OGB series or Fluo series, and are excited too close to the ANG-2 peak to separate the sodium and calcium signals. Recently, a series of long wavelength calcium indicators has become available (mostly from AATBioquest). One advantage of these new indicators is that that their excitation maxima are far enough towards the red end of the spectrum that there is minimal contamination between the two indicators when ANG-2 (for sodium) is excited at 513 nm. One good indicator in this series is Cal-630, which is best excited at 607 nm (Figure 8A). For our experiments we use either 595 nm (the peak of the closest available LED) or 561 nm (the closest available laser line). We also designed custom dichroic and emission filters for this pair of indicators with notches near 595 nm or 561 nm to pass the excitation light with either the LED or laser as we did for the other indicator pairs (Miyazaki and Ross, 2015). Using Cal-630 instead of bis-fura-2 gives good calcium signals (Figure 8A) with less background. An additional advantage of this and analogous indicators (e.g., Calbryte-630) for future work is that uncaging experiments can be combined with simultaneous sodium and calcium measurements since there is no need to excite near 375 nm, a wavelength region commonly used to uncage glutamate or other bioactive compounds. Using bis-fura-2 or SBFI would require excitation at this wavelength, which would prevent combining the measurements with uncaging.

One concern with using combinations of indicators is that there might be some contamination of one signal by the other if there is overlap in the excitation spectra. For the bis-fura-2:ANG-2 combination or the SBFI:OGB-1 combination there is almost no overlap in the excitation spectra (Miyazaki and Ross, 2015). In addition, their changes are in the opposite directions for parallel increases in ion concentration. However, when using the ANG-2 and Cal-630 combination there is more concern since the fluorescence changes are in the same direction and there is a small amount of Cal-630 excitation at the ANG-2 excitation peak (520 nm, Figure 8A, top). To investigate the Cal-630 fluorescence contamination we measured the signal from a bAP with 520 nm and 595 nm LEDs in a cell filled only with Cal-630 (Figure 8A, top). The Cal-630 signal from 520 nm excitation was 29% of the signal from 595 nm excitation. But it is easy to correct for this contamination, since it is always the same percentage of the signal at the preferred wavelength (Figure 8A). Figure 8B shows an experiment where we applied this correction procedure to the sodium signal measured at 520 nm. In this case the correction was small because the change in fluorescence of Cal-630 was smaller than the ANG-2 fluorescence. In other experiments (not shown) the correction was larger but still quantifiable. This correction procedure is similar to the way the spectra of chemical mixtures are separated into their components in some forms of spectroscopy.

## Discussion

In this article we reviewed our approach to high speed simultaneous sodium and calcium imaging. The original system (Miyazaki and Ross, 2015) using multiplexed LEDs was good enough to detect sodium and calcium signals from dendritic spines in rat hippocampal pyramidal neurons (Miyazaki and Ross, 2017). Building on this system we added spot illumination with lasers. This laser system has improved S/N, less background light and less photodynamic damage. One limitation of this system is that the spot illumination, which can vary from 2.5 μm to 30 μm diameter (depending on the fiber size and the objective lens) does not cover an extensive dendritic region. In this case the experiments may miss details about the spatial extent of synaptic activation on a particular dendrite or if more than one dendrite participates in the response. However, the system is designed in a way that it is easy to switch between LED and laser excitation even while maintaining electrical recording. It can also do a combination of one LED and one laser, which may be advantageous, for example, if the sodium signal is from a focal synaptic site and the calcium signal is from a propagating calcium spike.

We also introduced a new pair of indicators for simultaneous sodium/calcium imaging. This pair (ANG-2, Cal-630) does not require excitation near 380 nm as the previous pairs (bis-fura-2/ANG-2 and SBFI/OGB-1) require. Using this new pair should reduce background autofluorescence and photodynamic damage since both of these problems are greater at shorter wavelengths. In addition, uncaging stimulation (using laser pulses at 379 nm) can be incorporated into the system with no change in hardware.

One property of the laser excitation system, which might be of some value in future experiments, is that the lasers can be pulsed at very high rates, up to 200 MHz, with pulse widths fully under computer control. This property may provide the basis for more patterned excitation that may optimize the S/N of the measurements while minimizing photodynamic damage.

## Author Contributions

KM, JL and WR conceived experiments. KM, performed experiments. KM and WR analyzed experiments and wrote manuscript.

## Conflict of Interest Statement

The authors declare that the research was conducted in the absence of any commercial or financial relationships that could be construed as a potential conflict of interest.

## References

[B1] Acker C. D. Hoyos E. Loew L. M. (2016). EPSPs measured in proximal dendritic spines of cortical pyramidal neurons. eNeuro 3:ENEURO.0050-15.2016. 10.1523/eneuro.0050-15.2016 27257618 PMC4874537

[B2] Lee P. Yan P. Ewart P. Kohl P. Loew L. M. Bollensdorff C. (2012). Simultaneous measurement and modulation of multiple physiological parameters in the isolated heart using optical techniques. Pflugers Arch. 464, 403–414. 10.1007/s00424-012-1135-6 22886365 PMC3495582

[B3] Miyazaki K. Ross W. N. (2015). Simultaneous sodium and calcium imaging from dendrites and axons. eNeuro 2:ENEURO.0092-15.2015. 10.1523/eneuro.0092-15.2015 26730401 PMC4699831

[B4] Miyazaki K. Ross W. N. (2017). Sodium dynamics in pyramidal neuron dendritic spines: synaptically evoked entry predominantly through AMPA receptors and removal by diffusion. J. Neurosci. 37, 9964–9976. 10.1523/jneurosci.1758-17.2017 28904093 PMC5637120

[B5] Popovic M. A. Carnevale N. Rozsa B. Zecevic D. (2015). Electrical behaviour of dendritic spines as revealed by voltage imaging. Nat. Commun. 6:8436. 10.1038/ncomms9436 26436431 PMC4594633

[B6] Tanese D. Weng J.-Y. Zampini V. De Sars V. Canepari M. Rozsa B.. (2017). Imaging membrane potential changes from dendritic spines using computer-generated holography. Neurophotonics 4:031211. 10.1117/1.nph.4.3.031211 28523281 PMC5428833

[B7] Vogt K. E. Gerharz S. Graham J. Canepari M. (2011). High-resolution simultaneous voltage and Ca^2+^ imaging. J. Physiol. 589, 489–494. 10.1113/jphysiol.2010.200220 21115640 PMC3055538

